# Filipino nurses’ experiences and perceptions of the impact of climate change on healthcare delivery and cancer care in the Philippines: a qualitative exploratory survey

**DOI:** 10.3332/ecancer.2023.1622

**Published:** 2023-11-09

**Authors:** Mary Anne Tanay, Josie Quiambao-Udan, Oliver Soriano, Genevieve Aquino, Paula Melizza Valera

**Affiliations:** 1 Faculty of Nursing, Midwifery and Palliative Care, King’s College London, SE1 8WA London, UK; 2 University of Santo Tomas, Manila 1008, Philippines; 3 Lancashire and South Cumbria NHS Foundation Trust, PR5 6AW Lancashire, UK; 4 Global Climate Policy, Manila 1000, Philippines; 5 Global Mental Health and Medicine, Manila 1000, Philippines; a https://orcid.org/0000-0002-3637-6742

**Keywords:** qualitative, climate change, Philippines, cancer, nurses, nursing, typhoons, natural disasters

## Abstract

**Background:**

Because of its geographical location, the Philippines is vulnerable to the effects of climate change and almost all types of natural hazards such as typhoons, earthquakes, and volcanic eruptions. Cancer is one of the leading causes of death in the Philippines and is one of the major public health concerns. Little is known about how climate change affects cancer services in the Philippines. As the biggest workforce in most institutions, having awareness and knowledge about disaster preparedness and management among nurses can help in reducing the devastating effects of natural disasters on health services. Thus, it is important to understand Filipino nurses’ experiences and perception of the impact of climate change on healthcare delivery and cancer care in the Philippines.

**Aim:**

This study explored Filipino nurses’ experiences and perception of the impact of climate change on healthcare delivery and cancer care in the Philippines.

**Methods:**

This is a descriptive qualitative exploratory study. Participants were recruited using the snowballing technique and completed an online survey. Forty-six nurses who were working in Luzon, Philippines at the time of the data collection were included in the analysis. Data were analysed using thematic analysis.

**Findings:**

Three themes were identified, namely: (1) effects of climate change causing disruption and delay in provision of patient care, (2) impact of climate change on nurses and a deep sense of duty, and (3) perceived impact on patients with cancer.

**Conclusion:**

Our study findings contribute to the existing literature that focuses on the impact of climate change-related events such as typhoons and floods on healthcare services and nursing staff. Several areas of cancer care are also impacted, particularly delays in treatment such as chemotherapy. Despite the challenges, the nurses in our study demonstrated a deep sense of commitment in carrying out their roles.

## Background

The United Nations defines climate change as ‘long term shifts in temperatures and weather patterns’ which are directly attributed to the increase of global temperatures because of cumulative and historical carbon emissions since the Industrial Revolution [23]. In the Philippines, scientists believe climate change is evidenced by an increase in intensity and frequency of typhoons resulting in flooding, landslides, and erosions [22]. Five of the ten deadliest typhoons of the Philippines between 1947 and 2014 have occurred between 2006 and 2016. More recently, and referred to as Super Typhoons, Haiyan (2013), Mangkhut (2018), Goni (2020) and Rai (2021), with speeds starting 157 mph and above, resulted in deaths and extensive infrastructure damage. For example, 6,300 individuals died due to typhoon Haiyan; more than 4 million people were displaced, and damages reached $2 billion [22]. Similarly, rising mean temperatures results in extreme heatwaves and droughts, affecting both marine and terrestrial environments.

The Philippines is identified as having the second largest number of people affected by natural disasters globally [3]. As an archipelago situated in the Pacific Ocean, the country is one of the most vulnerable not only due to extreme weather patterns but also by the rising sea level. In this context, the general Philippine population experiences recurrent natural disasters such as typhoons, flooding and earthquakes, impacting on each individual’s physical and mental health [19]. The effects of natural disasters can lead to a state of despair and shock which brings about symptoms of severe stress, and feelings of grief and sadness for prolonged periods [16]. The timely provision of healthcare services, including mental health, is vital in such situations.

Cancer is one of the leading causes of death in the country and is one of the major public health concerns [12]. Oncological care requires intensive capacity building and facility development to enhance the delivery of care [4]. In 2019, the National Integrated Cancer Control Act has been signed into legislation, pushing for reforms in developing cancer centres across geographical regions; educational enhancements for health human resources, and improving financial support mechanisms for cancer care [8]. However, the country faces barriers with the majority of cancer treatment institutions centred in the urban capital of Metro Manila [21], and the shortage of oncologists and oncology nurses, making cancer care continuously fragmented [10] and catastrophically expensive [17]. There are limited opportunities for specialised and advanced cancer nursing roles in the Philippines. Nurses receive minimal introductory oncology education and clinical skills when they start in a cancer unit and subsequently learn on the job [2]. How natural disasters and climate change affect cancer services in the Philippines is unexplored. The Philippines is among the countries worst affected by displacement due to natural disasters [11]; this is clearly a challenge for chronic conditions like cancer, which often requires long-term and multi-modal series of treatments.

Filipino nurses perceived they were lacking in disaster preparedness and limited in their awareness of workplace protocols for disaster management [14]. A prepared workforce is key for maintaining stability in care delivery to meet the needs of people [1]. As one of the first responders and the biggest workforce in most health institutions, having awareness and knowledge about disaster preparedness and management among nurses can help in reducing the devastating effects of natural disasters and increasingly frequent extreme weather events on health services [14]. Thus, it is important to understand nurses’ experiences and perceptions of climate change and related events. Further, we are also interested in understanding the nurses’ views on the impact of climate change on cancer care. Thus, this study aimed to explore Filipino nurses’ experiences and perception of the impact of climate change on healthcare delivery and cancer care in the Philippines.

## Methods

This descriptive qualitative exploratory study involved an online survey conducted in October 2022 through MSForms^TM^. Participants were eligible if they were registered Filipino nurses currently working in the Philippines at the time of the survey, regardless of their geographic location or field of nursing. Purposive sampling and the snowballing technique were used to identify participants. Key colleagues known to the researchers, including those who work in cancer centres, were approached who then shared the survey link with their registered nurse colleagues.

Study information was provided to participants on the survey landing page; consent was implied upon completion of the survey. Information about participants such as years of nursing experience, area of nursing expertise and location were obtained. Three broad questions were asked i.e., ‘Do you agree or disagree that climate change affects your work as a nurse and delivery of healthcare in your area?’, ‘Have you experienced a natural disaster caused by climate change in the last 3 years? Describe how this affected your work or life as a nurse’, and ‘If you are providing care for patients who are diagnosed with cancer, how do you think climate change affects their interventions and treatment?’.

**Figure 1.**
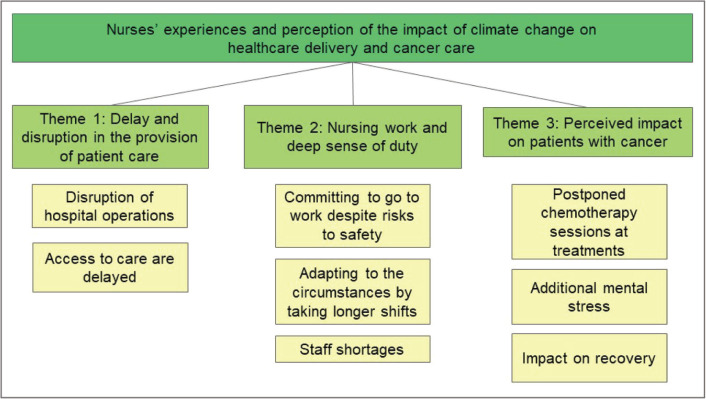
Thematic map.

Thematic analysis was used to analyse qualitative data. Mary Tanay and Paula Melizza Valera independently read and familiarised themselves with the raw data and generated data codes. Themes were identified then compared to develop initial themes. Discussions were conducted amongst all co-authors to refine and finalise themes. Figure 1 shows a thematic map of the study.

The final paper was reviewed and approved by all authors. Four co-authors are healthcare professionals; one is a global energy and climate policy expert. All authors have first-hand experience of typhoons and other natural disasters in the Philippines. The study was granted ethical clearance by King’s College London Research Ethics Committee (Reference: MRM-22/23-34234).

## Findings

**Figure 2.**
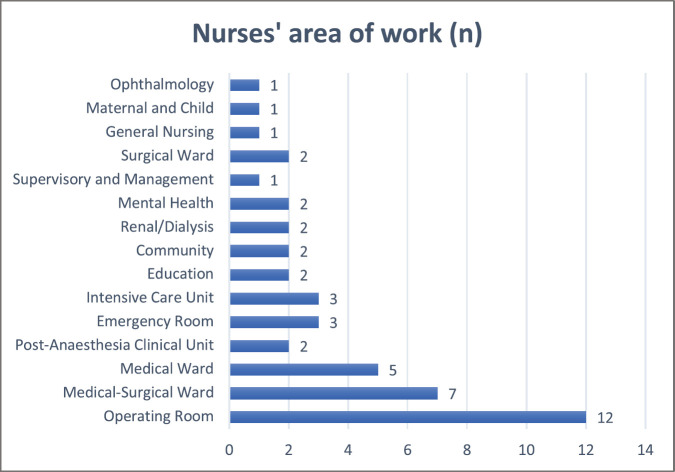
Area of work of participants.

Fifty Filipino nurses completed the survey; most participants were from Luzon (*n* = 46) and the rest from Visayas (*n* = 3) or Mindanao (*n* = 1). As practices can be different from these three main Philippine regions due to local culture, governance, and geographical influence, only responses of participants from Luzon (*n* = 46) were included in data analysis. The average number of years of clinical experience of the participants is 10 years. Although 30 nurses mentioned they were involved in providing care for patients affected by cancer, no participant identified their workplace as a cancer unit. Table 1 summarises participants’ demographic profile. Figure 2 shows an overview of the area of work of the nurse participants.

Three broad themes were identified in the analysis namely (1) effects of climate change cause disruption and delay in provision of patient care, (2) impact of climate change on nurses and a deep sense of duty, and (3) specific impact on patients with cancer. These themes are presented below with exemplar quotes from participants.

### Theme 1: Effects of climate change cause disruption and delay in provision of patient care

Climate change has been described by the nurse participants as frequent experience of ‘*unprecedented rainfall’* (R8), ‘*floods’* (R12), *‘typhoons’* (R5, R7) and *intolerable heat* (R11). For 20 nurses, climate change being experienced as heavy monsoon rains, typhoons and massive flooding create disruptions and delays in the delivery and provision of patient care, particularly those that entail face-to-face consultations and procedures.

*‘The sudden change in weather can delay your way of giving immediate care to your patients’* (R10). A community nurse has expressed ‘*Yes, typhoons are typical occurrences in our region, which also cause flooding and landslides. These hazards require us to provide additional services to the community but also hamper our capacity to deliver these services’* (R12).

These can be more disproportionately felt in inaccessible areas *‘When the weather seems unpredictable, the delivery of medical services to far-flung areas are affected’* (R49). Whilst online or virtual consultations have been an alternative,* ‘Delivery of care has been difficult reaching out the most deprived patients, and an online consultation is different from a face-to-face consultation’* (R2).

### Theme 2: Impact of climate change on nurses and a deep sense of duty

Thirty-nine nurses agreed climate change affected their work as a nurse and delivery of healthcare; six did not concur while one replied ‘*it depends on the facility*’ (R41) but did not give an explanation. The impact of weather-related events such as typhoons and floods on nursing education and nursing services were noticeable. There were ‘*suspension of classes and clinical shifts in hospital’* (R2) which ‘*affect the learning, health and safety of nursing students*’ (R3).

Nurses have also expressed their worry of being unsafe and the imminent risks of going to work even though there is an ongoing severe weather disturbance, *‘You already know that there are floods outside; your concentration is already divided’* (R47). Despite the risks, nurses still find the means to go to work, even if their safety is in question.

*‘I experienced typhoon. One of the barriers that affect me is transportation. Typhoons make vehicles stranded… But what I did was to walk from the area were the bus stop then crossed the area where there was a tricycle waiting. It made me feel nervous that time because the wind was so strong that any second, tragedy can happen but thank God I still managed to attend to my patients and survived my duty’* (R48).

Staff shortages were common due to additional adjustments the staff must make when parts of the hospital were flooded; this was made worse by the surge of patients coming in after a period of inactivity due to road closures. One may not be able to go to work because of the immediate consequences of the weather disturbance, *‘If there is a typhoon we cannot come to work because there is no available transportation’* (R5), ‘*When there is flooding, I cannot go to work*’ (R22). Hospitals can become short-staffed because of these conditions, forcing nurses to take on multiple shifts as the incoming nurses cannot get to work on time or were directly hit by the events.

*‘It was hard because we had to go on straight duty since nobody can relieve us at work’* (R50).

*‘I was on duty during the Ondoy (Ketsana) typhoon in Marikina. I was on duty in the Emergency Room. The flood reached the first floor of the hospital which forced our hospital to close for a few hours, then when we reopen waves of patients came by. We were short staffed due to other nurses were also trapped or affected by disaster, we the remaining staff were forced to work more than 24 hours’ *(R7).

Furthermore, nurses felt the quality of care can be compromised because nurses were tired resulting from working very long shifts or being stranded in flooded streets.

*‘…if an area is flooded during a typhoon, it may delay the healthcare service. And if there is a flooded area, some co-workers are unable to go to work…Which will force the previous shift to extend their duty in which affects delivery of healthcare because staff are exhausted’* (R40).

Despite the challenges of coming to work amid extreme weather conditions, the nurses showed commitment and a deep sense of duty to their community and the people they served. Interestingly, four out of six nurses who did not agree climate change affected them suggested nurses should be *‘able to adopt and be flexible’* (R36) and *‘prepared for any changes in the environment’* (R21).

*‘We go to work whatever the circumstances’* (R37).

*‘As a full pledge and a dedicated nurse, we didn't see that climate change affects our profession. Because we deliver our services to the people and to the community that we serve with compassion without thinking what will happen…as long as we know that we attended every individual who needs our services’* (R48).

### Theme 3: Specific impact on patients with cancer

Thirty of the study participants said they were involved in providing cancer care and services. They identified three main areas in which climate change can impact cancer care and patients with cancer. Firstly, disruptions caused by extreme weather result in the delay in delivery and patient access to cancer prevention, detection and management. Several nurses mentioned delays in chemotherapy due to typhoons and flooding. They also highlighted the effects of delays on patient outcomes and additional costs borne by the patient.

*‘Chemotherapy is a scheduled treatment for multiple months or weeks, so if there are natural disasters this could not only delay treatment schedule but also can be additional cost for the patient’* (R7).

*‘She is struggling with colorectal cancer stage 4. Her treatment was postponed due to continuous typhoon last 2020 in Bulan, Sorsogon and also COVID at that time. If there are no restrictions at that time and there are no destruction to roads everywhere she might have been able to have treatment as early as possible’* (R8).

*‘Some patients may have problems getting access to medications they need, and other patients may not be able to store their medications at the temperature required to preserve maximal efficacy’* (R48).

Hospital facilities can also be flooded, becoming unsafe for both staff and patients, and had led to evacuations*. ‘During typhoon Ursula (Phanfone), our hospital was one of the facilities that was destroyed by the typhoon. We needed to evacuate the patients and put to the safer place. All our (hospital) records were washed out by the typhoon’* (R39).

Secondly, participants emphasised the additional mental stress extreme weather events cause patients who are already going through a difficult time dealing with the cancer diagnosis and effects of treatments. Typhoons can destroy people’s homes and livelihood which can be mentally distressing.

*‘Seeing or witnessing a strong typhoon hitting your home will cause trauma, especially those people who have a critical illness, it may trigger their emotional state which can lead to another health issue if not treated well’* (R11).

Finally, participants also focused on impact of recovery of cancer patients who are already *‘immunocompromised’* (R24) and are more *‘vulnerable to illnesses’* (R25). Air pollution, water pollution and less access to fresh food such as those living in cities can make recovery slower. Extremely hot temperatures can cause *‘lack of sleep and rest’* (R35).

*‘The patient will be deprived with the basic human necessities such as clean air, fresh water, and healthy environment. These aspects are important to a cancer patient especially they are prone to infections due to their low immune system’* (R27).

## Discussion

Interestingly, despite several components of climate change such as temperature increase, altered rainfall pattern, sea-level rise, ocean acidification, extreme weather events, and climate-active pollutants, climate change in the Philippines has been mainly associated by the nurse participants with events such as unprecedented rainfall, floods, typhoons, and intolerable heat. This may be explained by the direct and profound impact of these events on nurses on a regular basis. Further, nurse responses were mainly focused on the disruptive impact of flooding from heavy rains and typhoons on hospital operations and delivery of care. These events certainly exacerbate existing socio-economic and health vulnerabilities in the society. Natural disasters aside, the Philippine health system already faces challenges in its pluralism, information asymmetry, fragmentation between private and public health, and disproportionate costs of care, pushing families into impoverishment [9]. Despite the decentralisation of the public health system on local governments [15], the unequal and uneven growth and development across geographical areas in the Philippines make health service delivery and financing more difficult for lower-income municipalities [5, 6]. Thus, vulnerabilities in the health sector are amplified during and after each landfall of a super typhoon. Delivery of medical services are likewise facing increased pressure due to diminished access of logistics and public transportation networks in both urban and rural landscapes. Telemedicine, as an approach for the continuity of care has proliferated and has become more acceptable and utilised by Filipino health providers, families and their patients beginning COVID-19, and was even considered ‘revolutionary’ [24]. Whilst telemedicine and consultations entail certain benefits, access to such mechanisms remains disproportionate and inequitable for the poor, with majority of its providers require out-of-pocket payments [5, 6]. Natural disasters and extreme weather conditions may also amplify the challenges of delivering services due to the lack of available community-based mental health services and a challenged workforce that requires training in disaster nursing and mental health care.

Unsurprisingly, nurses in our study highlighted how they have been directly affected by the consequences of harsh weather interruptions, particularly by heavy flooding. Similar to earlier research, our study showed problems with accessibility which led to nurses being unable to go to work and hindered delivery of care [7]. Heavy flooding creates a double impact on the nursing workforce. Firstly, incoming nurses are already tired prior to the start of their shifts due to challenges coming to work despite unfavourable weather conditions or flooded roads. Secondly, nurses who are already at work had to extend their working hours whilst waiting for the incoming staff and can also face the same challenges going home at the end of work. Tiredness can result in reduced cognitive and physical abilities [20], thus can compromise patient safety as the risk of making mistakes, errors and accidents is higher.

The safety of nurses is also put at risk or likely to be compromised as they attempt to continue to go to work, even if the conditions can be perilous. Indeed, there are a number of nurse participants who have expressed that climate change will not affect their job, as they will continue to perform their nursing duties. While this positive outlook is commendable, staff also suffer from devastating weather-related events [7] and have their own needs to address. It can be very challenging to provide care when nurses are themselves worrying about their livelihoods, homes and families. Support for nurses during periods of typhoons especially when there is a high risk of flooding is important. Aside from being provided temporary shelter [7], robust debriefing procedures across the clinical team may help to maintain team functioning.

Disrupted access to cancer care due to typhoons and flood-related events was highlighted by many nurse participants in this study. Typhoons cause loss of electricity, damaged or blocked roads, and disruptions in transportation; recovery from the devastation may take several days or weeks depending on severity. With an already fragmented cancer care service [10], any delay in screening and diagnosis can be detrimental to patient outcomes. Many nurses in the study mentioned delays in vital chemotherapy treatment schedules. For patients with cancer, the impact of typhoons on access to cancer services can mean the difference between survivorship or dying from the disease [18]. The effect of cancer can make patients with cancer less resilient against the effects of climate change-related events [13]. Indeed, a diagnosis of cancer can be a life-changing situation affecting physical, psychological, social, financial and spiritual aspects. In a country where healthcare is not readily available and free, many patients with cancer can experience hardship and financial toxicity. Those who provide treatment should consider offering patients psychological interventions such as psychosocial support, debriefing and cognitive behavioural therapy [19]. Factors identified by nurses which may contribute to slow recovery among cancer patients include air and water pollution as well as access problems to food and medicines when roads are blocked or damaged, particularly in the major cities. For example, Metro Manila already has a significant risk of flooding throughout the rainy months; these floods are made worse and more alarming by frequent typhoons. Air and water pollution can exacerbate not only physical problems but may increase risk for acquiring infectious diseases which can be life-threatening for patients with compromised immune system.

Our study findings contribute to the existing literature (as above) that focuses on the impact of climate change on healthcare services, nursing staff and cancer care. The growing literature on this topic may guide employers and policy makers when developing interventions for supporting nurses to effectively carry out their work during periods of severe weather conditions and flooding. Further, it can also inform strategies aimed at motivating nurses on undertaking further learning on disaster preparedness and management. Whilst many of the nurses’ perceptions and experiences in our study may be similar to experiences of other healthcare workers in the Philippines, future research should focus on healthcare teams which include other members of the multidisciplinary team such as doctors, community health workers, and allied health professionals to obtain wider and interdisciplinary perspectives on the impact of climate change on cancer care as a whole. Further, experiences of the effects of climate change among Filipino patients with cancer and their caregivers should be explored. More work should be done to reduce the impact on care for chronic conditions requiring multiple interventions such as cancer and the provision of mental health support to patients.

## Limitations

There are several limitations in this study. Although open-ended questions were used for the study, it was not possible to explore the responses further as responses were collected online. This may have affected the richness and depth of data collected. Further, most of the responses were from nurses based in Luzon, the largest island group in the country. Experiences of nurses from other parts of the country i.e., Visayas and Mindanao regions, particularly those from smaller islands, and geographically detached areas may be different thus should be explored further. It is worth noting that many nurses who participated in this study mentioned they were involved in providing cancer care. However, respondents did not identify themselves as ‘cancer or oncology specialist nurses’ nor that they were working in ‘oncology units’. This may reflect local models of care delivery in which nursing specialisms are scarce or limited [2]. Future work should focus on Filipino nurses who identify themselves as cancer specialist nurses. Due to the short duration of data collection, potential participants may not have had the chance to answer the survey. We also acknowledge the small study sample; findings from this exploratory qualitative study may inform future research in this important topic.

## Conclusion

Our study findings contribute to the existing literature that focuses on the impact of climate change on healthcare services, nursing staff and cancer care. Nurse participants in this study recognised the disruptive impact of flooding from heavy rains and typhoons on hospital operations. Nurses are also directly affected by the consequences of harsh weather interruptions, with nurses reporting unsafe travelling conditions and difficulty going to work thus hindering delivery of care. Several areas of cancer care are also affected, particularly delays in treatment such as chemotherapy. Despite challenges due to climate change-related events, nurses showed a deep sense of commitment to fulfilling their roles.

## Conflicts of interest

## Funding

## Author contributions

## Figures and Tables

**Table 1.** Participant demographic details.

Participant study identifier	Region	Years of clinical experience	Area of work
R1	Luzon	20	Education
R2	Luzon	24	Maternal and child
R3	Luzon	27	Education
R4	Luzon	7	Operating room
R5	Luzon	9	Operating room
R6	Luzon	5	Medical ward
R7	Luzon	11	Medical-surgical ward
R8	Luzon	5	General nursing
R9	Luzon	25	Supervisory and management
R10	Luzon	10	Medical-surgical ward
R11	Luzon	5	Medical ward
R12	Luzon	7	Community
R13	Luzon	11	Community
R14	Luzon	12	Operating room
R15	Luzon	29	Mental health
R16	Luzon	10	Post-anaesthesia clinical unit
R17	Luzon	30	Medical-surgical ward
R18	Luzon	3	Operating room
R19	Luzon	1	Medical ward
R20	Luzon	6	Medical-surgical ward
R21	Luzon	26	Operating room
R22	Luzon	1	Operating room
R23	Luzon	5	Medical-surgical ward
R24	Luzon	4	Operating room
R25	Luzon	2	Operating room
R26	Luzon	7	Surgical ward
R27	Luzon	0.5	Post-anaesthesia clinical unit
R28	Luzon	2	Medical-surgical ward
R29	Luzon	19	Operating room
R30	Luzon	10	Operating room
R31	Luzon	10	Operating room
R34	Luzon	5	Ophthalmology
R35	Luzon	30	Medical ward
R36	Luzon	7	Operating room
R37	Luzon	3	Mental health
R38	Luzon	12	Renal/Dialysis
R39	Luzon	8	Emergency room
R40	Luzon	5	Surgical ward
R41	Luzon	1	Emergency room
R42	Luzon	1	Medical ward
R43	Luzon	12	Emergency room
R45	Luzon	13	Intensive care unit
R47	Luzon	8	Intensive care unit
R48	Luzon	7	Medical-surgical ward
R49	Luzon	10	Intensive care unit
R50	Luzon	7	Renal/Dialysis
